# Thirty years of Ca^2+^ spark research: digital principle of cell signaling unveiled

**DOI:** 10.52601/bpr.2024.240031

**Published:** 2024-10-31

**Authors:** Fujian Lu, Pengcheng Yang, Donghui Zhang, Xianhua Wang, Heping Cheng

**Affiliations:** 1 Department of Cardiology, Zhongshan Hospital, Institutes of Biomedical Sciences, Fudan University, Shanghai Institute of Cardiovascular Diseases, Shanghai 200032, China; 2 State Key Laboratory of Biocatalysis and Enzyme Engineering, School of Life Science, Hubei University, Wuhan 430062, China; 3 National Biomedical Imaging Center, State Key Laboratory of Membrane Biology, Peking-Tsinghua Center for Life Sciences, College of Future Technology, Peking University, Beijing 100871, China

**Keywords:** Ca^2+^ signaling, Ca^2+^ microdomain, Digital system design

## Abstract

Calcium ions (Ca^2+^) are an archetypical and most versatile second messenger in virtually all cell types. Inspired by the discovery of Ca^2+^ sparks in the 1990s, vibrant research over the last three decades has unveiled a constellation of Ca^2+^ microdomains as elementary events of Ca^2+^ signaling and, more importantly, a digital-analog dualism as the system design principle of Ca^2+^ signaling. In this brief review, we present a sketchy summary on advances in the field of sparkology, and discuss how the digital subsystem can fulfill physiological roles otherwise impossible for any analog system. In addition, we attempt to address how the digital-analog dualism endows the simple cation messenger with signaling speediness, specificity, efficiency, stability, and unparalleled versatility.

## INTRODUCTION

Intracellular Ca^2+^ signaling is paradoxically simple and complex. One divalent cation connecting thousands of target proteins (or Ca^2+^ signalome), Ca^2+^ emerges as the most versatile intracellular messenger orchestrating a myriad of physiological processes from muscle contraction to neuronal firing, and from cell proliferation to apoptosis (Berridge *et al.* 2000, 2003; Bootman 2012). Yet, its powerful information-coding ability derives almost entirely from the binding and unbinding of this cation to its proteinaceous effectors as well as the electrical currents it generates when moving across a biological membrane. The enigma arises as to how Ca^2+^ orchestrates molecular players to fulfill remarkably diverse and sometimes opposing physiological functions within a given cell.

**Figure 1:**
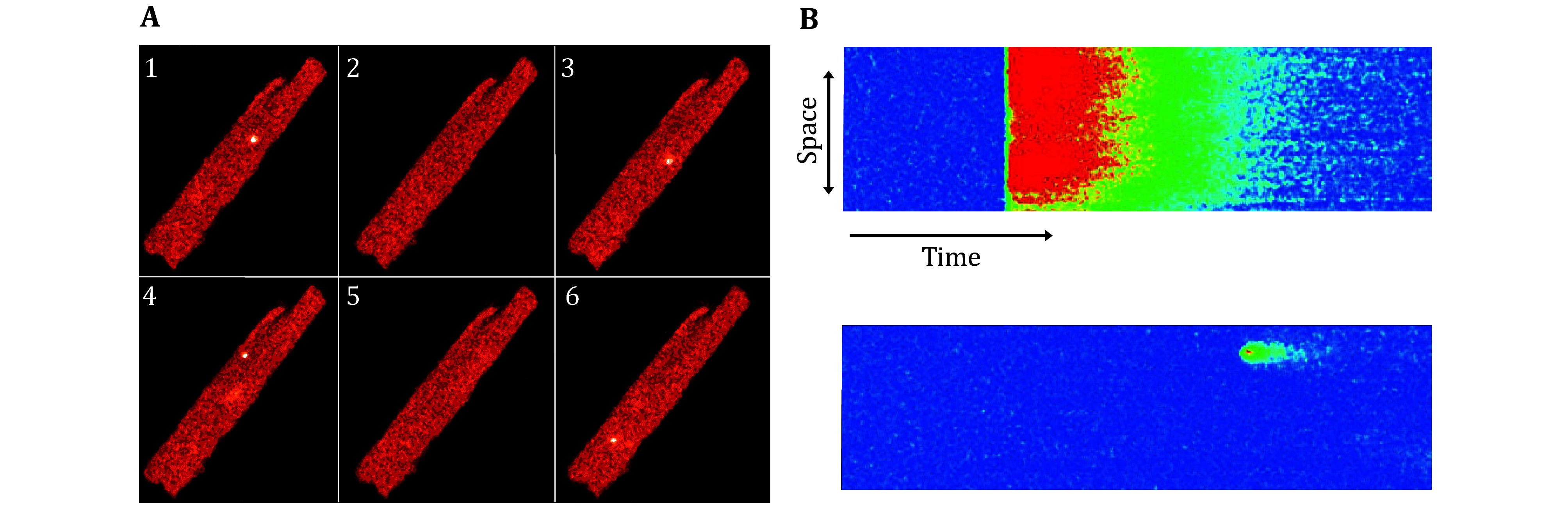
Ca^2+^ sparks. **A** Two-dimensional confocal images of Ca^2+^ sparks in a quiescent cardiac myocyte (scan rate 1.0 s/frame). **B** Line-scan confocal images of an action potential-elicited [Ca^2+^]_i_ transient (top) and a spontaneous spark (bottom) (scan rate 2.0 ms/line). Time and space ordinates are displayed in the horizontal and vertical directions, respectively (Cheng *et al.* 1993)

First discovered in 1993 with the advent of confocal microscopy and fast, high-contrast fluorescent probes, such as Fluo3 (Kao *et al.* 1989), Ca^2+^ sparks (Fig.1) represent discrete, local and brief events elemental to intracellular Ca^2+^ signaling in the heart (Cheng *et al.* 1993). Since then, vibrant research in muscles, neurons, and other excitable and non-excitable cells has unveiled a large family of spark-like elemental Ca^2+^ signaling events, an exquisite constellation of their subcellular organization, and unique and unexpected signaling modalities enabled by such Ca^2+^ microdomains. These advances have led to the appreciation of the digital-analog dualism of the Ca^2+^ signaling system (Cheng and Lederer 2008), with the digital subsystem encompassing all discrete, all-or-none signaling microdomains exemplified by Ca^2+^ sparks, gaining teleological insights into diverse supramolecular Ca^2+^ signaling substructures found in virtually all types of cells. In this brief review, based on the 2023 Bei Shizhang award lecture delivered by the senior author, we attempt to elucidate the digital building principle of the Ca^2+^ signaling system. Through a few examples, we discuss how the digital-analog dualism may unify the simplicity and the versatility of Ca^2+^ signaling.

## Ca^2+^ SIGNALING MICRODOMAINS

In multicellular organisms, Ca^2+^ concentrations in the extracellular space and membrane-bounded intracellular Ca^2+^ stores, primarily the endoplasmic reticulum (ER), are in the millimolar (mmol/L) range. However, sustained high Ca^2+^ levels in the cytosol are extremely toxic, so resting cytosolic Ca^2+^ must be maintained at low levels, typically in the range of tens to a few hundreds of nmol/L, implicating a 10,000-fold gradient across the plasma membrane and the membrane of the Ca^2+^ stores. Signaling involves change, and the steep gradients are exploited to produce rapid, high Ca^2+^ signals by mobilizing Ca^2+^ from these reservoirs to the cytosol, coupled with re-sequestration mechanisms to timely terminate the signal. Ca^2+^ ions are impermeable to lipid biomembranes, and their fast-rate transmembrane mobilization is mediated by various Ca^2+^ channels that are gated by physical factors such as membrane voltage, mechanical stretch and temperature; ligands including neurotransmitters, inositol 1,4,5-trisphosphate (IP_3_), ryanodine, and Ca^2+^ itself; and even ER store depletion. Transmembrane Ca^2+^ mobilizers also include Ca^2+^ pumps and exchangers, which operate at a slower rate (on a molecule-to-molecule comparison). While channel-mediated Ca^2+^ flux always moves down-hill the electrochemical potential gradients of Ca^2+^, transporters can facilitate up-hill Ca^2+^ movement at the cost of free energy input. From a cytosol-centric perspective, each Ca^2+^ mobilizer acts as a Ca^2+^ source when it injects Ca^2+^ flux into the cytosolic compartment; and as a Ca^2+^ sink when it removes Ca^2+^ ions to other membrane-partitioned compartment. In the cytosol milieu rich with Ca^2+^ binding sites, the bound-to-free Ca^2+^ ratio reaches about 100. Ca^2+^ diffusion is slowed by an order of magnitude, and the spatial range of Ca^2+^ signaling is also locally confined. This allows Ca^2+^ signals at a few μm distances to operate quasi-independently. From the source-sink viewpoint, a Ca^2+^ binding site (or Ca^2+^ buffer) can function as both a source and a sink, depending on when and where it discharges or mops up free Ca^2+^ ions. Furthermore, there exist many nanoscale and micro-scale membranous substructures, in the form of membrane-apposed cleft, button, vesicle, cistern, tubule, filopodium and lamellipodium, *etc*. In such subspaces, Ca^2+^ handling is greatly influenced by physical constraints imposed by the membrane boundaries.

In the common-pool model of intracellular Ca^2+^ signaling, cytosolic Ca^2+^ is theorized to be spatially homogenous, and all effectors are governed by the same intracellular Ca^2+^ dynamics (Stern 1992). However, the very existence of discrete Ca^2+^ sparks that span only ~2 μm diameter and last about 20 ms (Cheng and Lederer 2008) has provided direct evidence that this is a significant oversimplification. In mammalian ventricular myocytes, cardiac dyads are supramolecular nanoscopic substructures formed by apposition of transverse (T) tubules (*i*.*e*., invaginations of the plasma membrane) and cisterns of the ER/sarcoplasmic reticulum (SR) (Franzini-Armstrong *et al.* 1999; Lu and Pu 2020). Excitation-contraction (E-C) coupling links membrane depolarization to myocyte contraction, which occurs primarily at cardiac dyads via the Ca^2+^-induced Ca^2+^ release (CICR) mechanism (Fabiato and Fabiato 1972; Fabiato *et al.* 1972). Specifically, membrane depolarization opens voltage-gated L-type Ca^2+^ channels (LTCCs) on the T-tubules, and resultant Ca^2+^ influx rushes into the dyadic cleft and serves as the activator of an array of type 2 ryanodine receptor (RYR2) Ca^2+^ release channels, liberating SR store Ca^2+^ and giving rise to Ca^2+^ sparks. In a typical cardiac myocyte, a constellation of Ca^2+^ sparks arising from ~10,000 dyads summates to produce a cell-wide Ca^2+^ transients that activates cell contraction. Essentially, cardiac E-C coupling reflects the behavior of a digital system in which Ca^2+^ sparks serve as the elementary units. These unitary Ca^2+^ sparks are locally controlled and digitally activated, providing a teleological explanation for salient architectural features such as the nanoscale dyadic cleft and microscale spatial separation of dyads, which align with the similarly sized length of a sarcomere. This alignment, possibly not coincidental, matches the dimension of sparks.

**Figure 2:**
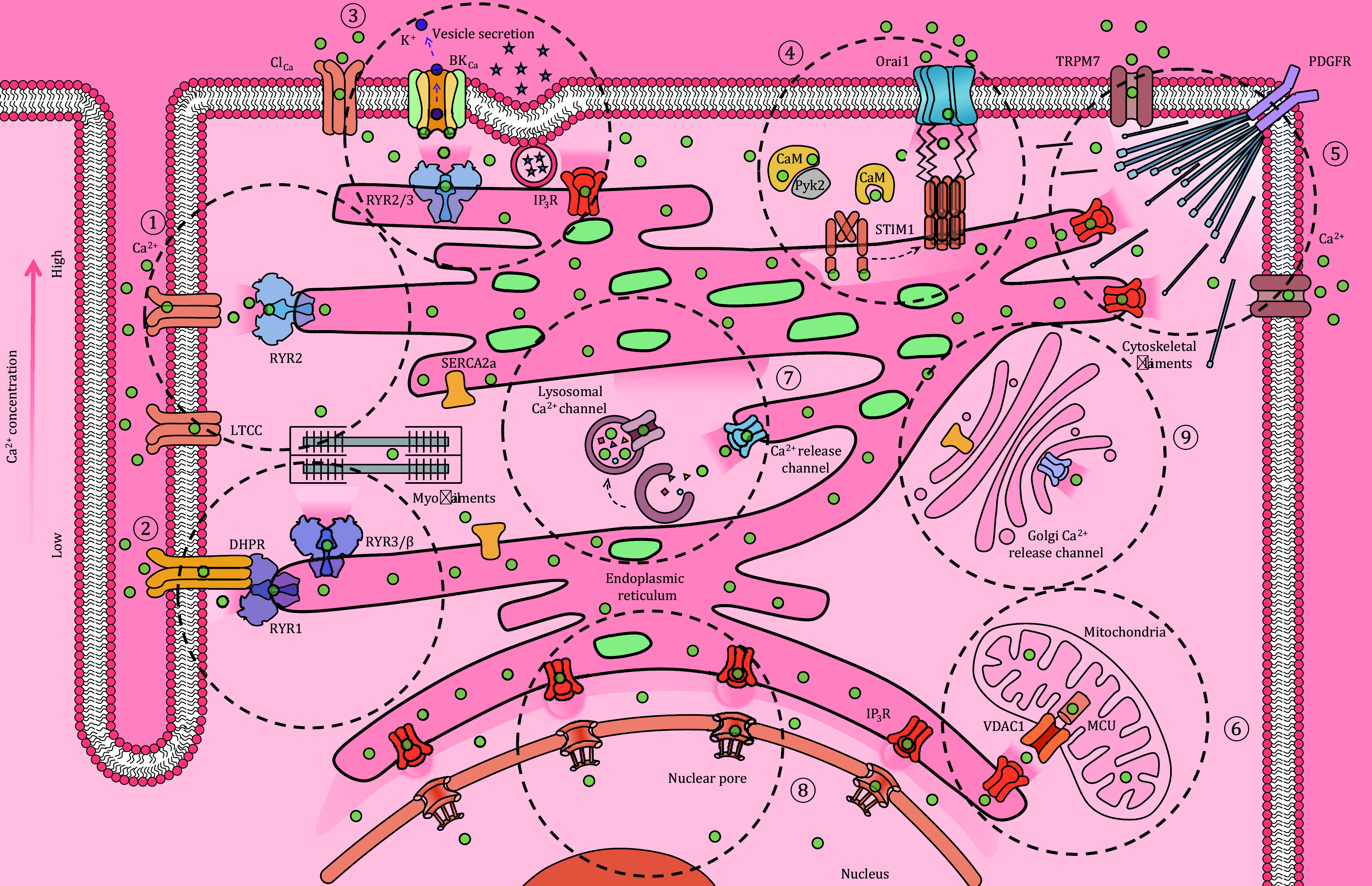
Exemplary Ca^2+^ signaling microdomains. Cartoon illustration of various microdomains featured in different types of cells, all being artificially integrated in a hypothetical single cell. (1) Cardiac dyad. Single LTCC openings generate Ca^2+^ sparklets to activate a RYR2 Ca^2+^ nanospark in the cleft and a Ca^2+^ spark of 2 μm diameter in the surrounding space, as well as a Ca^2+^ blink in the connected ER/SR cistern. (2) Skeletal muscle triad (half structure illustrated). Arrayed RYR1 is mechanically gated by DHPR acting as the voltage sensor to initiate SR Ca^2+^ release, which, in turn, may activate nearby RYR3/β array via the CICR mechanism. (3) Subsurface cistern, to support subsurface sparks seen in smooth muscle cells and DRG neurons. (4) Clustered Orai1 and STIM1 forming an elemental unit for SOCE. Its digital operation gives rise to Ca^2+^ glows which morphology and kinetics are shaped by membranous structures like invadopodia. (5) Multimodal Ca^2+^ signaling microdomain. In human lung fibroblasts, Ca^2+^ flickers arise from both TRPM7 Ca^2+^ influx, sensing mechanical signals, and IP_3_R Ca^2+^ release, sensing chemotactic signal mediated by platelet-derived growth factor receptor (PDGFR) signaling pathway, and dynamically regulate the assembly and disassembly of cytoskeletal filaments, and thereby steer the direction of cell migration. (6) Mitochondria-associated membranes (MAMs), the contact sites between mitochondria and ER, are a central hub for Ca^2+^ signaling, apoptosis, autophagy and lipid biosynthesis. (7) ER surface Ca^2+^ microdomains, sites of phase separation of protein complex for specification of autophagosome initiation. (8) Perinuclear Ca^2+^ waves from the ER in the nuclear envelope, with high Ca^2+^ passing through the nuclear pores. (9) Golgi Ca^2+^ microdomain

The digital mode of Ca^2+^ signaling is universal to virtually all cell types examined, and a family of elementary Ca^2+^ signaling events has been characterized in both excitable and non-excitable cells (Fig.2). In particular, Ca^2+^ sparks are present in skeletal (Baylor *et al.* 2002; Hollingworth *et al.* 2001; Klein *et al.* 1996; Tsugorka *et al.* 1995) and smooth muscle myocytes (Burdyga and Wray 2005; Nelson *et al.* 1995; ZhuGe *et al.* 1998), neuroendocrine cells (*e*.*g*., chromaffin cells) (ZhuGe *et al.* 2006), and neurons containing different isoforms of RYRs (De Crescenzo *et al.* 2004; Koizumi *et al.* 1999; Ouyang *et al.* 2005). In the nanoscale dyadic cleft, a Ca^2+^ nanospark with a higher Ca^2+^ level has been visualized by using dyad-targeted Ca^2+^ sensors GCaMP6f-Triadin1/Junctin (Shang *et al.* 2014). Ca^2+^ sparklet arising from a plasmalemmal LTCC (Wang *et al.* 2001) or TRPV4 channel (Sonkusare *et al.* 2012) has been optically recorded in cardiac or smooth muscle cells, respectively. Inside the Ca^2+^ store, local depletion of Ca^2+^ occurs concurrently during a Ca^2+^ spark, and this phenomenon of Ca^2+^ depletion has been visualized as a Ca^2+^ blink (Lu *et al.* 2020), and is thought to provide a possible mechanism to signal spark termination (Brochet *et al.* 2005) and local activation of store-operated Ca^2+^ entry (SOCE), manifested as Ca^2+^ glows in the invadopodium of cancer cells during metastasis (Lu *et al.* 2019). In oocytes, discrete local Ca^2+^ release of IP_3_ receptor (IP_3_R) origin gives rise to Ca^2+^ puffs (from clustered IP_3_Rs) and blips (from single IP_3_Rs) (Lock *et al.* 2019; Yao and Parker 1994). In human lung fibroblasts, discrete, local and short-lived high Ca^2+^ microdomains, namely Ca^2+^ flickers (Wei *et al.* 2009), are representative of a converging multimodal signaling. The source Ca^2+^ of a flicker comes from both TRPM7, which senses plasmalemmal mechanical stress, and IP_3_R, which is co-activated by local Ca^2+^ and IP_3_, the latter being linked to extracellular chemotactic signals. More recently, it has been shown that high Ca^2+^ microdomains on the ER surface trigger liquid-liquid phase separation to specify autophagosome initiation sites (Zheng *et al.* 2022). Perinuclear Ca^2+^ waves in the nuclear envelope (Luo *et al.* 2008), with high Ca^2+^ passing through the nuclear pores, can preferentially access the matrix of the nucleus and regulate Ca^2+^-dependent biological processes within. The Golgi apparatus serves also as a reservoir for Ca^2+^ in the cell. Ca^2+^ release from the Golgi Ca^2+^ channel can be triggered by various signaling pathways and stimuli, and create local high Ca^2+^ signals influencing cellular responses such as secretion and cell growth (Pizzo *et al.* 2011). Overall, it is safe to conclude that each cell type harbors unique nanoscale supramolecular substructures that support dynamic microdomain Ca^2+^ signaling. Their location and mode of action are tailored to the specific needs of vital physiological functions.

## DIGITAL PRINCIPLE OF Ca^2+^ SIGNALING

The universality of Ca^2+^ microdomains and their unique mode of action have led to the appreciation of a digital-analog dualism for cell Ca^2+^ signaling (Cheng and Lederer 2008), in which discrete Ca^2+^ signaling microdomains form a digital subsystem that is intertwined with an analog subsystem comprising Ca^2+^ signals in the bulk cytosol. Specifically, the digital subsystem is built for high-threshold Ca^2+^-dependent processes, such as activation of BK_Ca_ and Cl_Ca_, activation of CaMKII and calcineurin, and vesicle secretion (Ouyang *et al.* 2005), all of which require Ca^2+^ levels to reach tens of μmol/L and beyond. This subsystem is discontinuous in space and individual microdomains operate intermittently in an all-or-none fashion. Its overall Ca^2+^ signaling effect is thus depicted by a digital function, *S*(*n*, *t*_event_), where *n* and *t*_event_ denote the number and timing of microdomain activation. Spatially and temporally patterned activation of the digital subsystem may enable intracellular Ca^2+^ signaling to achieve roles that are otherwise impossible for any analog system. In the following we briefly discuss sub-principles of digital Ca^2+^ signaling as illustrated by examples in different types of cells or physiological contexts.

### Linear and stable control of an inherently non-linear unstable system

CICR implies positive feedback and an inherently strong non-linearity, best suited for signaling rapidity and high-gain amplification. Theoretically, when the gain of CICR is greater than one, a common-pool system would become unstable such that the system as a whole operates in the all-or-none regime (Stern 1992). In fact, as informed from the whole-cell Ca^2+^ transient and contraction strength, cardiac E-C coupling driven by CICR is gradable and tightly controlled by the duration and the magnitude of the triggering LTCC Ca^2+^ current, creating the so-called CICR paradox. In the digital Ca^2+^ signaling regime, however, the paradox is readily resolved. At the system level, global Ca^2+^ transient is, to the first order approximation, a linear function of the number of the unitary Ca^2+^ sparks evoked. The positive feedback and high-gain amplification are largely confined within individual dyads. Nevertheless, instability does occur when a cardiac myocyte is challenged with Ca^2+^-overloading conditions. In this scenario, E-C coupling is no longer graded by LTCC triggering Ca^2+^, Ca^2+^ sparks tend to ignite neighboring dyads, and regenerative inter-dyadic CICR would result in propagating Ca^2+^ waves that are highly arrhythmogenic (Cheng *et al.* 1996).

### Enhanced efficiency and specificity of Ca^2+^ signaling

This sub-principle applies to all Ca^2+^ signaling processes whose effects depend hyper-linearly on Ca^2+^ concentration. Given the same total Ca^2+^ mobilized, spatial and temporal condensation would disproportionally enhance their signaling efficiency. For instance, in a process with a power of two Ca^2+^-dependence, the overall effect would be augmented as much as 100 times, if its spatial and temporal dimensions are each compressed by a factor of 10 (assuming effectors are uniformly distributed spatially). In this regard, we have shown that about seven subsurface Ca^2+^ sparks suffice to trigger a vesicle release in DRG neurons (Ouyang *et al.* 2005), while the amount of Ca^2+^ involved could barely raise Ca^2+^ level by a few nmol/L if uniformly distributed.

An additional advantage of a digital organization is related to signaling specificity. This is because the signal is turned on only at precise locations with specific timing. Ample examples can testify to this point. During melanoma invasion, SOCE-mediated Ca^2+^ glows promote CaM/Pyk2 aggregation and matrix metalloproteinase secretion in invadopodia (Lu *et al.* 2019). High Ca^2+^ on the ER surface can selectively activate phase separation of FIP200 for specification of autophagosome initiation sites on the ER (Zheng *et al.* 2022). By confining the high-Ca^2+^ process at sites of MAMs (Vance 2014), inter-organelle Ca^2+^ signaling can be channeled without affecting effectors in the cytosol.

### Microdomain Ca^2+^ fulfills roles distinct from that of global Ca^2+^

An elegant example came from the study of Ca^2+^ spark regulation of vascular tone. In arterial smooth muscles, while global Ca^2+^ elevation signifies muscle contraction, subsurface Ca^2+^ sparks relax the blood vessels. Mechanistically, high subsurface Ca^2+^ activates high-threshold BK_Ca_ to cause membrane hyperpolarization, shut-off of LTCC Ca^2+^ influx and eventual decrease of bulk cytosolic Ca^2+^ concentration (Nelson *et al.* 1995).

### Complex behavior emerged from coordinating microdomain Ca^2+^ activity

In migrating cells, Ca^2+^ plays multifunctional roles in directional sensing, cytoskeleton redistribution, traction force generation, and relocation of focal adhesions. Multimodal Ca^2+^ signaling microdomains are present at the front of migrating fibroblasts, with TRPM7 sensing mechanical signals and PDGFR sensing gradients of chemotactic signals. These signals converge onto plasmalemmal TRPM7- subspace IP_3_R clusters to generate Ca^2+^ flickers. Spatial and temporal coordination of these digital events by both mechanical and chemotaxis signals is thought to orchestrate the assembly and disassembly of cytoskeletal filaments and thus direct cell migration (Wei *et al.* 2009).

## NEW HORIZONS

Unraveling Ca^2+^ signaling microdomains and the digital-analog dualism represents a paradigm shift in our understanding of the design principle of cellular Ca^2+^ signaling system as a whole. Deeper insights into Ca^2+^ signaling in both physiology and pathophysiology have also been obtained through elucidating molecular mechanisms, physiological significance, and pathological implications of Ca^2+^ microdomains. However, many important unmet challenges lie ahead. First, the assembly of Ca^2+^ microdomains remains poorly understood. We are only beginning to appreciate the complex mechanism underlying the formation and positioning of the supramolecular assembly of Ca^2+^ microdomains. In heart, how do dyads as the hallmark architecture establish and maintain their striking registration along with sarcomeric Z-lines? Our recent work on *in situ* and *in* *vivo* proteomic mapping of dyadic proteins revealed that CMYA5, a master protein localized to Z-lines, contributes to tethering junctional SR (jSR) adjacent to these structures. T-tubules subsequently form and co-localize with jSR, yielding organized, properly positioned dyads (Lu *et al.* 2022), in which junctophilin family proteins play important roles (Hall *et al.* 2024). Second, at the system level, matching a plethora of Ca^2+^ signalome proteins of different Ca^2+^ affinity and functionality with a constellation of Ca^2+^ microdomains of distinctive characteristics must be a daunting task, letting alone the combinatorial interaction among them. Developing innovative approaches to systematically elucidate the sorting mechanism and its underlying cell logic would yield profound insights into the digital-analog dualism of cell signaling. Third, there are urgent needs to discriminate dysregulation of microdomain Ca^2+^ signaling in a wide range of diseases (Gomez *et al.* 1997; Song *et al.* 2006). Advanced imaging techniques, such as three-dimensional electron microscope reconstruction and super-resolution light microscopy, coupled with new generations of microdomain-targeted probes, offer powerful means to uncover new architectures, new roles and new modes of microdomain Ca^2+^ signaling *in vitro* and *in vivo*, as well as their subtle changes in pathological conditions. In addition, computational modeling approaches are also valuable in integrating quantitative cell Ca^2+^ data into a unifying framework.

Given the archetypical role of Ca^2+^ as a second messenger, future research should systematically investigate whether the digital principle is applicable to other common biological messengers such as cAMP, IP_3_, nitric oxide, and reactive oxygen species (ROS). In this regard, previous studies have shown that cAMP signaling is highly compartmentalized during β_2_-adrenergic stimulation (Kuschel *et al.* 1999; Mika *et al.* 2012) and that cAMP signaling microdomains selectively modulate intra-dyadic, but spare extra-dyadic targets including the Ca^2+^ pump SERCA2a in cardiac myocytes (Zhou *et al.* 2009). As another example, single-mitochondrion ROS bursts during multi-faceted signaling events known as “mitoflashes” (Wang *et al.* 2008) operate in a frequency-dependent, digital mode, and play important roles in the regulation of cardiac ATP homeostasis (Wang *et al.* 2017), synaptic plasticity (Fu *et al.* 2017), and brown adipocyte thermogenesis (Chen *et al.* 2024). Altogether, we are witnessing the emergence of a fundamental principle of cell physiology: the digital organization of signaling microdomains is crucial for achieving rapid, specific, efficient, stable, and unparallelly diverse cell signaling.

## Conflict of interest

Fujian Lu, Pengcheng Yang, Donghui Zhang, Xianhua Wang and Heping Cheng declare that they have no conflict of interest.
